# Nanofocusing of the free-space optical energy with plasmonic Tamm states

**DOI:** 10.1038/srep39125

**Published:** 2016-12-20

**Authors:** Linyu Niu, Yinxiao Xiang, Weiwei Luo, Wei Cai, Jiwei Qi, Xinzheng Zhang, Jingjun Xu

**Affiliations:** 1 The MOE Key Laboratory of Weak-Light Nonlinear Photonics, TEDA Applied Physics Institute and School of Physics, Nankai University, Tianjin 300457, China; 2 Synergetic Innovation Center of Chemical Science and Engineering, Tianjin, 300071, China

## Abstract

To achieve extreme electromagnetic enhancement, we propose a plasmonic Tamm states (PTSs) configuration based on the metal-insulator-metal Bragg reflector, which is realized by periodically modulating the width of the insulator. Both the thick (2D) and thin (3D) structures are discussed. Through optimization performed by the impedance-based transfer matrix method and the finite difference time domain method, we find that both the electric field and magnetic field intensities can be increased by three orders of magnitude. The field-enhancement inside the PTSs configuration is not limited to extremely sharp waveguide terminal, which can greatly reduce processing difficulties.

Surface plasmon polaritons (SPPs), the electromagnetic waves coupled to charge excitations at the surface of metal, have been widely studied due to potential applications in photonic technologies^1^,^2^,^3^. The most prominent features of SPPs are their ability to localize light beyond the diffraction limit and their huge electromagnetic enhancement, both of which are vital for the development of enhancing nonlinearities^4^,^5^, surface-enhanced Raman scattering^6^ and surface-enhanced fluorescence^7^. Compared with nanoantennas^8^, which have to be made into arrays because of their small interaction area, plasmonic waveguides have advantages to collect the free-space light efficiently. Numerous plasmonic devices have been proposed theoretically or demonstrated experimentally, such as tapered metal V-grooves^9^ with a field intensity enhancement factor of about 130, and tapered metal-insulator-metal (MIM) waveguides^10^ with an intensity enhancement factor of 400 by focusing light into a 14 × 80 nm^2^ area. However, for these proposals, their impressive maximum field enhancements were always achieved by waveguides with sharp tapered ends. The 3D structural fabrication of tapered waveguides is cumbersome in the sub-10-nm region and the field enhancement can be weakened due to nonlocal effects^11^,^12^.

Plasmonic Tamm states (PTSs)^13^, a newly discovered type of Tamm states, have been proposed as an effective solution to produce strong field enhancement. Unlike optical Tamm states (OTSs, also known as Tamm plasmons)^14^ that can be excited directly by both TE- and TM-polarized waves in an optical system, PTSs exist in a plasmonic system and can only be excited by TM-polarized waves. When PTSs are generated, fields are resonantly enhanced at the interface between the MIM Bragg reflector (BR) and the metal according to the phase-matching condition: *r*_M_*r*_BR_ = 1, where *r*_M_ is the reflection coefficient of SPPs by the metal and *r*_BR_ is the one by the BR. Compared with previous light-focusing configurations, PTSs have several advantages: (1) PTSs provide nearly two extra orders of magnitude for the intensity enhancement in addition to the dimension confinement, and (2) the enhancement of PTSs is not based on decreasing the cross-section of the waveguide, a process that would increase production difficulty. Nonetheless, the originally proposed MIM BR for the PTSs was achieved by periodic modulation of the dielectrics in the MIM waveguide, which was complex and difficult process. Moreover, only very thick (2D) structure was discussed while the practical thin (3D) structure had been poorly studied.

In this work, PTSs configuration is proposed which periodically modulates the widths of MIM waveguides. Both 2D and 3D structures of this waveguide are studied. Compared with the one made by periodic modulation of dielectrics, this new structure can be fabricated through electron beam or focused ion beam direct-writing lithography, avoiding complex fabrication procedures like filling the MIM waveguides with different dielectric materials. The new structure also provides a more efficient field enhancement.

## Results

**Figure 1:**
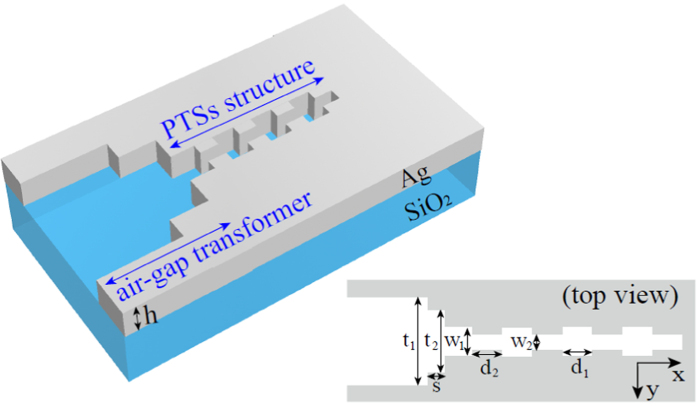
The 3D generation configuration of PTSs including an air-gap transformer. The configuration is composed of an air-gap transformer and a PTSs structure on a silica substrate. The inset shows a top view of the structure.

The scheme of the 3D PTSs generation configuration including a transformer is illustrated in Fig. 1. The free-space light is efficiently coupled into the PTSs configuration in the form of SPPs through the transformer. Multiple configurations have been designed to achieve the efficient coupling between the free-space light and SPPs modes, such as the quarter-wave transformers^15^,^16^, air-gap couplers^17^ and multi-section microcavities^18^. In this paper, the air-gap transformer is chosen for its simplicity and convenience. All the waveguide cores are filled with air (refractive index *n*_0_ = 1). The structure is fabricated on a silica substrate with a constant refractive index *n* = 1.45. The metal is silver, whose permittivity is characterized by the well-known Drude model 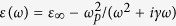, where *ε*_*∞*_ = 3.7, *ω*_*p*_ = 9.1 eV, and *γ* = 0.018 eV. This dielectric function fits well with the experimentally determined value in the visible and infrared frequency regions^19^.

According to the transmission line theory, the characteristic impedance of MIM waveguide can be deduced from the voltage and current in the waveguide. For the fundamental mode, the corresponding impedance is expressed as^20^:


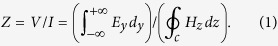


When the thickness of the waveguide in the *z*-direction is very thick, for instance, we etch the structure on bulk metal for hundreds of micrometers or even more, the PTSs structure in this case can be treated as a 2D model, the impedance is written as^21^:


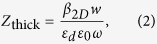


where *w* is the width in the *y*-direction, *ε*_*d*_ is the permittivity of the dielectric material, *ω* is the angular frequency of the electromagnetic field and *β*_2*D*_ is the 2D propagation constant along the *x*-direction. *β*_2*D*_ can be solved directly from the dispersion relation: −tanh(*k*_*d*_*w*/2) = *k*_*m*_*ε*_*d*_/*k*_*d*_*ε*(*ω*), where *k*_*m*_ and *k*_*d*_ denote the corresponding transverse propagation constants. They are related to the propagation constant *β*_2*D*_ as 
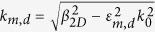
, where *k*_0_ is the propagation constant in vacuum. Similarly, the impedance for thin MIM waveguide in our impedance model is expressed in an approximate form:


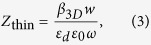


where *β*_3*D*_ is the 3D propagation constant that can be solved numerically. The validity of *Z*_thin_ will be proved by comparing the impedance model with full-wave numerical simulations.

### 2D plasmonic Tamm states design/test scenario

**Figure 2:**
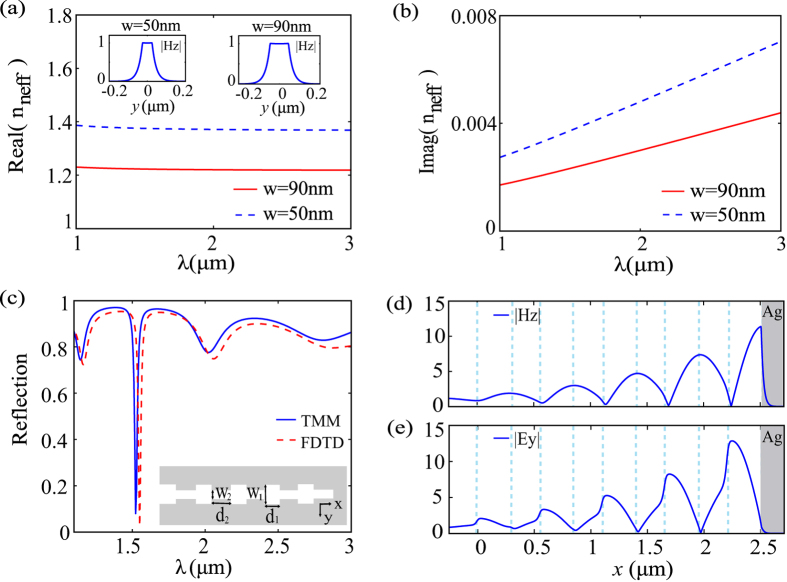
The dispersion relation, reflection spectra and normalized amplitude distributions along the center axis for the 2D PTSs configuration. (**a**) Real and (**b**) imaginary parts of the effective indices for the 2D MIM waveguides. Inset: Magnetic field distributions of the fundamental modes along the *y* axis. (**c**) Reflection spectra calculated by the TMM (solid blue line) and FDTD (dashed red line). (**d**,**e**) The normalized distributions of |*H*_*z*_| and |*E*_*y*_| along the center axis.

In this section, the simplified 2D PTSs model is discussed for the thick waveguide. As depicted in the inset of Fig. 2(c), we take *w*_1_ = 90 nm and *w*_2_ = 50 nm. These dimensions are currently achievable with standard nanofabrication techniques such as electron-beam lithography, focused ion beam milling, and various etching processes. The operation wavelength is the telecom wavelength 1550 nm. The real and imaginary parts of the effective indices of the fundamental modes supported by the 2D MIM waveguides are shown in Fig. 2(a,b), and the insets depict the cross-sectional view of the magnetic field along the *y* axis. Based on the transmission line theory, the MIM waveguide terminal can be treated as an inductance *L*_*Ag*_^22^, of which the corresponding impedance is written as *Z*_*Ag*_ = −*iωL*_*Ag*_. Hence, we can simplify the maximization of field enhancement as a maximization of power delivery in an impedance, where the reflection minimum corresponds to the phase-matched wavelength. The optimization process to find a reflection minimum at 1550 nm is as follows: First, trial and error tests for *d*_*1*_ and *d*_*2*_ are calculated via the impedance based transfer matrix method (TMM) (see methods). Then, the time-consuming FDTD calculation is performed to determine the best values of *d*_*1*_ and *d*_*2*_ around the values found by the TMM. It has been shown that the TMM results match well with full-wave simulations^13^. In our case, as shown in Fig. 2(c), the subtle difference between the reflection spectra calculated by the TMM and FDTD originates from discontinuous interfaces, where extra phases are introduced because *w*_*1*_ and *w*_*2*_ are different.

One set of optimized parameters are found to be *d*_*1*_ = 252 nm and *d*_*2*_ = 300 nm for 5 periods. It should be noted that small numbers of periods are not able to confine enough energy at the BR terminal, and long MIM waveguides will introduce more Ohmic loss^13^. In Fig. 2(c), the reflection spectra calculated by the TMM (solid blue line) and FDTD (dashed red line) are plotted. As designed, the reflection reaches a minimum of about 2.6% at *λ* = 1550 nm in the spectrum calculated by FDTD. There is a blue-shift of about 25 nm in the results that calculated by TMM. In Fig. 2(d,e), the normalized distributions of |*H*_*z*_| and |*E*_*y*_| along the center axis of the PTSs configuration are presented. Magnetic and electric fields are both normalized. The dashed blue lines indicate the positions of discontinuous interfaces. It is obvious that both the electric and magnetic fields are confined and enhanced at the MIM BR terminal. The maximum |*H*_*z*_| occurs at the interface between the MIM BR and the metal with an enhancement factor of 11.4, and the maximum |*E*_*y*_| occurs around the first interface near the MIM BR terminal with an enhancement factor of 12.9.

**Figure 3:**
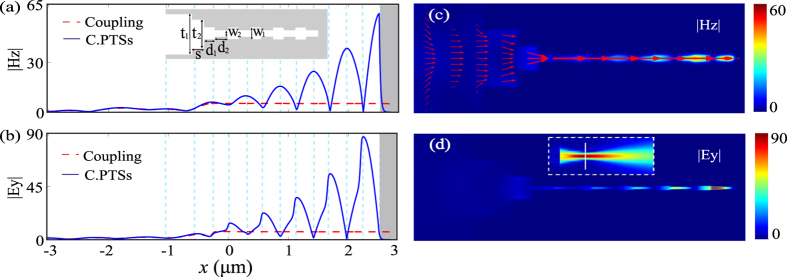
Normalized amplitude distributions for the 2D PTSs structure including the air-gap transformer. (**a**,**b**) Field amplitude distributions along the central axis for light propagating in an air-gap transformer in connection with the 90 nm-width waveguide (Coupling) and the PTSs structure (C.PTSs). (**c**,**d**) Field amplitude distributions in the *x−y* plane. The arrow surface indicates the Poynting vector. The inset of (**d**) indicates the Gaussian beam in the free space and the vertical white line indicates the entrance of the transformer.

Then, the coupling of free-space light is considered with regard to an air-gap transformer with a width larger than the diffraction limit, e.g. *t*_*1*_ = 1 *μ*m. The schematic of the simulated structure is presented in the inset of Fig. 3(a). The field amplitude |*H*_*z*_| of a Gaussian beam distributed in free space is shown in the inset of Fig. 3(d) and the vertical white line indicates the position of the entrance of the air-gap transformer. The Gaussian beam has a waist of 800 nm. The electromagnetic fields are normalized according to the Gaussian beam at 2 *μ*m on the left side of the waist along the central axis. The fields are enhanced by factors of 2.3 for |*H*_*z*_| and 2 for |*E*_*y*_| at the position of the waist in vacuum. The optimized parameters of the air-gap transformer are *t*_*2*_ = 400 nm and *s* = 260 nm, of which the coupling efficiency is 86.7% as calculated by FDTD. All parameters for the PTSs structure remain unchanged. For the whole structure, the normalized distributions of |*H*_*z*_| and |*E*_*y*_| along the center axis are presented in Fig. 3(a,b) for light propagating in an air-gap transformer in connection with the 90 nm-width waveguide (Coupling) and the PTSs structure (C.PTSs). It is clear that dual field enhancements have been realized: The first field enhancement with enhancement factors of 5.1 for |*H*_*z*_| and 6.7 for |*E*_*y*_| results from the efficient coupling of the air-gap transformer; the second enhancement arises from the resonance enhancement of PTSs. After this dual enhancement, the total maximum enhancement factors are 59.3 for |*H*_*z*_| and 87.1 for |*E*_*y*_| at the MIM BR terminal. The normalized amplitude distributions are plotted in Fig. 3(c,d). The arrow surface in Fig. 3(c) indicates the Poynting vector, where the length of the arrow stands for the logarithmic magnitude of the Poynting vector.

### 3D plasmonic Tamm states design/test scenario

**Figure 4:**
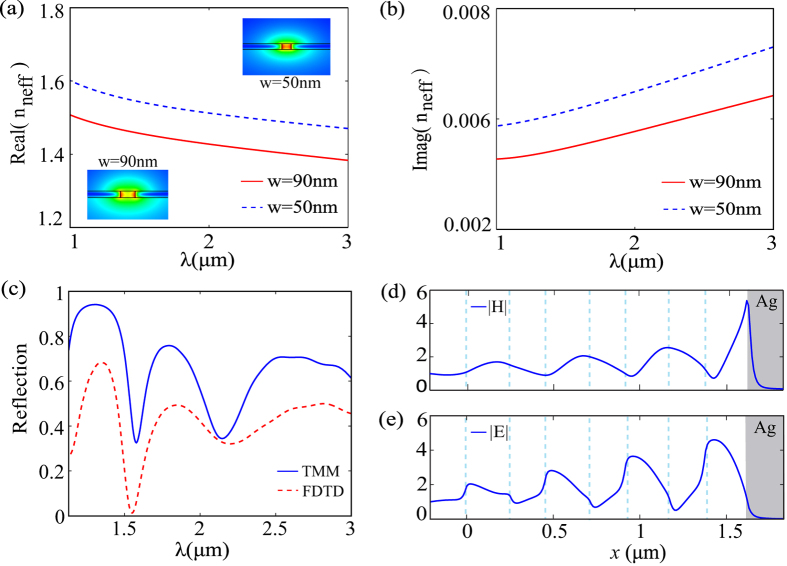
The dispersion relation, reflection spectra and normalized amplitude distributions along the center axis for the 3D PTSs configuration. (**a**) Real and (**b**) imaginary parts of the effective indices for the 3D MIM slot waveguides. Inset: Cross-sectional view of the magnetic field distribution. (**c**) Comparison of reflection spectra calculated by the TMM (solid blue line) and FDTD (dashed red line). (**d**,**e**) Normalized distributions of |*H*| and |*E*| along the center axis of the PTSs configuration as calculated by FDTD.

As discussed above, the 2D PTSs configuration has excellent properties for electromagnetic enhancement. However, in other applications, it is also important to confine the electromagnetic energy into a thin waveguide. As presented in Fig. 1, we assume that the waveguide has a thickness of *h* = 50 nm on a silica substrate. The parameters *w*_1_ = 90 nm and *w*_2_ = 50 nm remain unchanged, and a similar analysis is employed. The propagation constant *β*_3*D*_ is calculated via the finite element method (FEM). The effective indices of the fundamental modes supported by the 3D MIM waveguides are shown in Fig. 4(a,b). The insets provide a cross-sectional view of the magnetic field distributions. For wavelength *λ* = 1550 nm, the optimized parameters for PTSs are found to be *d*_*1*_ = 210 nm and *d*_2_ = 250 nm and the best period number is *N* = 4. As shown in Fig. 4(c) the comparison of the reflection spectra calculated by the TMM (solid blue line) and FDTD (dashed red line) are plotted. It is observed that the TMM results agree well with the spectrum calculated by FDTD, which proves the validity of the approximate 3D impedance in equation (3). The reflection measured by FDTD is lower than that by TMM, because the energy leakage is not taken into consideration in the TMM model. The normalized distributions of |*H*| and |*E*| along the center axis of PTSs configuration are shown in Fig. 4(d,e). The maximum enhancement factors are 5.5 for |*H*| and 4.6 for |*E*|, which are less compared with those in 2D waveguide. The capacity of the confinement is weakened here because the 3D MIM waveguide is an open system^23^.

**Figure 5:**
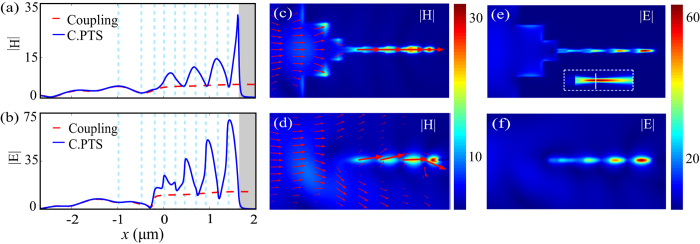
Normalized field distributions for the 3D PTSs structure including an air-gap transformer. (**a**,**b**) Field amplitude distributions along the central axis for light propagation in an air-gap transformer in connection with waveguide (Coupling) and the PTSs structure (C.PTSs). Field amplitude distributions in the *x−y* plane (**c**,**e**) and *x−z* plane (**d**,**f**). The arrow surfaces in (**c**,**d**) manifest the Poynting vector. The inset of (**e**) indicates the Gaussian beam in the free space.

Finally, taking the free-space coupling into consideration, an air-gap transformer is introduced with parameters *h* = 50 nm, *t*_1_ = 1 *μ*m, *t*_*2*_ = 400 nm and *s* = 270 nm. All parameters for the 3D PTSs structure remain unchanged. The Gaussian beam has a beam waist of 800 nm and the entrance of the transformer is positioned at the waist. The fields are enhanced by factors of 3.6 for |*H*| and 6.8 for |*E*| at the waist position in vacuum, which is larger than the 2D counterpart due to the additional dimension confinement in the *z* direction. After FDTD simulation, the coupling efficiency is obtained as 12.7% by collecting the energy flow in an area of 1200 × 200 nm^2^ in the *y−z* plane of the transformer. The reflection is 9.6%, so about 77.7% of the optical energy is leaked into the substrate or the free space. As shown in Fig. 5(a,b), the normalized distributions of |*H*| and |*E*| along the center axis of PTSs configuration are presented for light propagation in an air-gap transformer in connection with the 90 nm-width waveguide (Coupling) and the PTSs structure (C.PTSs). It is observed that |*H*| is enhanced by a factor of 5 and |*E*| by a factor of 12.3 when light is coupled into the MIM waveguide via the transformer. Then, considering the PTSs effect, the total enhancement factors are 30.8 for |*H*| and 63.6 for |*E*| at the MIM BR terminal. In Fig. 5(c–f), the distributions of |*H*| and |*E*| in the *x−y* plane and *x−z* plane are shown respectively. The arrow surfaces in Fig. 5(c,d) manifest the Poynting vector in the *x−y* plane and *x−z* plane. As in the case of 2D, the length of the arrow stands for the logarithmic magnitude of the Poynting vector. Although the confinement in the *x−y* plane is good as shown in Fig. 5(c), large amounts of energy is leaked into the air and substrate due to the poor confinement of the 50 nm structure in the *x−z* plane as shown in Fig. 5(d).

The parameter *h* used in the 3D geometry is as thin as 50 nm, which corresponds to a limiting case. Given that the energy confinement is good in the *x−y* plane while the leakage to the air and substrate is severe in the *x−z* plane, the most effective method for improving the coupling efficiency would be to enlarge the thickness of the film. Additionally, higher refractive index of the central region^23^ or another coupler in the *x−z* plane^10^ will increase confinement and hence the field enhancement.

## Discussion

A PTSs configuration is proposed by periodically modulating the width of the insulator in the MIM waveguide. In combination with the air-gap transformer, the free-space light can be efficiently coupled into the PTSs configuration. Both the thick (2D) and thin (3D) PTSs structures are designed and simulated with the help of the impedance-based TMM and FDTD, in which the 3D approximate impedance proves to be efficient. As for the 2D configuration, the total coupling efficiency between the free-space gaussian beam and the SPPs is 86.7% and the the total enhancement factors are 59.3 for |*H*_*z*_| and 87.1 for |*E*_*y*_|. The 50-nm thick 3D PTSs structure, while losing more energy that its 2D counterpart, is still able to enhance the |*H*| by 30.8 times and |*E*| by 63.6 times with a coupling efficiency of 12.7%. Compared with the conventional tapered-waveguide nanofocusing technique, which can only confine the optical energy in the dimension perpendicular to the propagation direction, the PTSs configuration adds the remaining dimension in the propagation direction for confinement. This air-gap PTSs structure has the advantage of convenient fabrication procedure, which makes it a promising optical device for integrated photonics and able to provide significant applications in increasing nonlinearities, surface-enhanced Raman scattering and surface-enhanced fluorescence.

## Methods

### Impedance-based transfer matrix method

The impedance-based transfer matrix method (TMM) is a powerful tool for the analysis of periodic structures^24^,^25^. Taking the boundary condition of the electromagnetic field at the interface *j* into account, the matrix (*M*_*j*_) representing the reflection and transmission at the discontinuities is defined as:


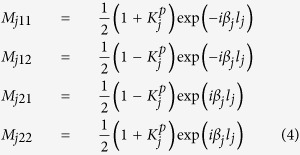


where *l*_*j*_ = *X*_*j*+1_ − *X*_*j*_, *X*_*j*_ and *X*_*j*+1_ are the positions of two nearest discontinuous interfaces between *j* and *j* + *1*. The parameter 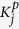 is defined as 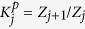, *Z*_*j*+1_ and *Z*_*j*_ are the impedances of region *j* + 1 and *j*, respectively. Combining the equations (2,3,4), the total reflection spectra can be acquired by 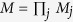, namely the reflections are given as *r* = *M*_21_/*M*_11_.

### Numerical simulations

The mode-solving approach for *β*_3*D*_ was calculated by using the FEM in the commercially available software package COMSOL MULTIPHYSICS. The reflection spectra and the near-field field distributions were calculated by FDTD simulations, using the commercial software package (Lumerical Solutions). The mesh spacing used in all simulations was 5 nm. And the structure was surrounded by perfectly matched layers in all directions.

## Additional Information

**How to cite this article**: Niu, L. *et al*. Nanofocusing of the free-space optical energy with plasmonic Tamm states. *Sci. Rep.* **6**, 39125; doi: 10.1038/srep39125 (2016).

**Publisher's note:** Springer Nature remains neutral with regard to jurisdictional claims in published maps and institutional affiliations.
